# Advanced breast cancer with cachexia: A case report

**DOI:** 10.1097/MD.0000000000024397

**Published:** 2021-01-29

**Authors:** Nan Liu, Shuting Li, Junmei Jia, Ying Qiao, Yuanfei Li

**Affiliations:** a Department of Oncology; b Department of Imaging, The First Hospital of Shanxi Medical University, Taiyuan, Shanxi, P.R. China.

**Keywords:** breast cancer, cachexia, muscle loss, radiological quantification, vertebral metastasis

## Abstract

**Rationale:**

Cachexia is a clinically relevant syndrome in cancer that is associated with reduced tolerance to anticancer therapy, reduced quality of life, and reduced survival rates. Cachexia is most prevalent in pancreatic, gastric, colorectal, lung, and head and neck cancers. It is rarely documented in breast cancer patients.

**Patient concerns:**

In our case report of a breast cancer patient with bone metastasis who was monitored throughout the course of her treatment, we document the development of cachexia using image analyses in relation to her metastatic burden. In the 2-year period, from April 10, 2015, to February 09, 2017, she lost 16% of her baseline weight. During this time, she was repeatedly hospitalized for chest tightness, edema of both lower limbs, numbness and pain in the left lower extremity and backache.

**Diagnoses:**

Our patient was a 46-year-old premenopausal woman when she was firstly diagnosed. Several years after surgery for invasive ductal carcinoma of the left breast, she had multiple systemic bone metastases (the thoracic spine, the ribs, etc), lung metastasis, bilateral axillary lymph node metastasis, and metastasis of the right neck lymph node in IV area.

**Interventions:**

The patient completed 6 cycles of postoperative adjuvant chemotherapy and long-term endocrine therapy after a radical mastectomy for breast cancer. During the fourth progression, 6 cycles of rescue chemotherapy were performed. Local lumbosacral radiotherapy, and lumbar surgery were carried out to relieve symptoms after several progressions.

**Outcomes:**

She became extremely thin, weighing only 50 kg at admission on July 23, 2018. This eventually led to multiple organ failure and death.

**Lessons:**

We noted a strong negative correlation between the abdominal muscle area and the metastatic tumor area at the second lumbar vertebral (L2) level. The monitoring of abdominal muscle wasting may serve as a marker, and therefore a prognostic factor, for both cachexia and the extent of metastatic disease. This is especially true with breast cancer, where metastasis to bone is frequent. Our data from a computational tomography radiological quantification, may provide clinicians with early indications of the extent of cachexia in metastatic breast cancer patients.

## 1 Introduction

Cancer cachexia is a metabolic syndrome characterized by anorexia, loss of weight, decreased muscle strength, and resistance to conventional nutritional support. Approximately 50% to 80% of patients with advanced cancer are diagnosed with cachexia, particularly those with metastatic disease.^[1,2]^ Over 20% of cancer patients die from cachexia. It is most common in cancers of the pancreas, stomach, colorectum, lungs, and head and neck,^[3]^ and has become increasingly common in breast cancer patients in recent years. The relationship between metastasis and cachexia in breast cancer patients may be more complex than previously thought.

Cachexia is responsible for the reduction in the quality and length of life of cancer patients. Bone is a preferred site for breast cancer metastasis, leading to pathologic bone loss due to increased osteoclast-induced bone resorption.^[4]^ Many biological factors, such as the Tumor Necrosis Factor-α produced by tumor cells, could attribute to proteolysis and skeletal muscle atrophy through protein synthesis inhibition and enhanced muscle degradation.^[5]^

The grade of malignancy of a tumor is primordially associated with both the development of metastatic lesions and cancer-associated cachexia, which are multifactorial conditions that depend on factors present in the micro- and macroenvironments of the tumor-bearing patient. We aimed to characterize the relationship between cancer progress and cachexia by studying metastatic breast cancer in a patient with typical cachexia syndrome. We found that an increased tumor burden in metastatic breast cancer patients was strongly associated with decreased muscle mass and weight loss.

## 2 Case summary

The patient was a 46-year-old premenopausal woman when she was firstly diagnosed, with a long course of disease. She underwent a radical left mastectomy in April 2009 due to the discovery of a mass in the left breast. Postoperative pathology: left breast invasive ductal carcinoma, no cancer observed in the tumor base, no cancer metastasis in the surrounding lymph nodes (0/19), and no cancer metastasis in the left axillary lymph nodes (0/6). Immunohistochemistry: estrogen receptor (ER) (++), progesterone receptor (-), human epidermal growth factor receptor 2 (-), epidermal growth factor receptor (-), kiel67 antigen (ki-67) positive cells greater than 5% (Table 1). Tumor node metastasis classification is pT2N0M0 (IIA phase). Postoperatively, a CAF regimen (Cyclophosphamide+ Doxorubicin+ Fluorouracil) was implemented for 6 cycles of regular chemotherapy, followed by Toremifene.

**Table 1:** Tumor immunohistochemistry. Tumor was biopsied at the primary site and at metastatic sites. The tumor was found to be invasive ductal breast carcinoma. See Figure 1.

Specimen	ER	PR	Her2	Ki67
Primary breast cancer	++	-	-	5%
Metastatic lymph node	+/-	-	+	

ER = estrogen receptor, Her2 = human epidermal growth factor receptor 2, Ki67 = kiel67 antigen, PR = progesterone receptor.

In July 2012, the patient was admitted to the hospital due to chest tightness and difficulty breathing. After the relevant examination, lung metastasis and multiple metastatic tumors of the thoracic vertebra were considered. In July 2014, she presented with mild edema of the face and lower limbs and was diagnosed with stable disease (SD). In November 2014, the patient's body was edema, her chest tightness was aggravated, and metastasis reached the ribs, sacrum, and bilateral ilium. In January 2015, she was admitted to the emergency department due to aggravation of chest tightness, and edema of the face and lower limbs. Karnofsky Performance Status: 20 points. The metastases were in the sixth thoracic vertebra (T6), T9, T11, right 5, 8 rib, L1 and L2. The patient's hormone levels were evaluated again: premenopausal (Table 2). The patient was administered with Exemestane+ Goserelin. Regular reviews over the following 10 months showed no tumor progression. Life quality improved significantly, Karnofsky Performance Status:80 to 90 points.

**Table 2:** Hormone levels in 2015. The patient's hormone level was evaluated again: premenopausal, which help select endocrine therapy drugs.

	E2 (less than73–147)	FSH (16.74–113.59)	LH (10.87–58.64)
October	10 pmol/L	9.88 IU/L	0.32 IU/L
November	79 pmol/L	9.70 IU/L	0.23 IU/L
December	4 pmol/L	10.14 IU/L	0.04 IU/L

E2 = estradiol, FSH = follicle -stimulating hormone, LH = leuteinizing hormone.

**Figure 1:**
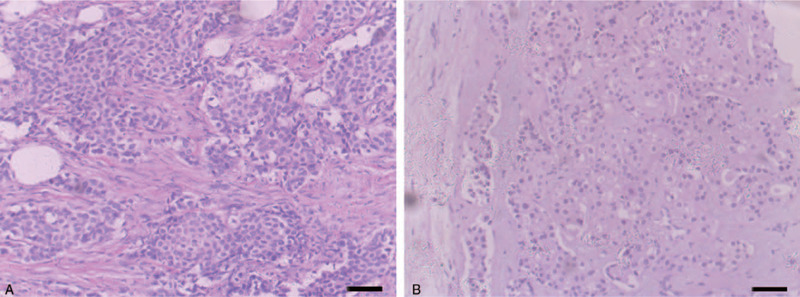
H&E images of breast tumor and cervical lymph node metastasis. (A) Shows invasive ductal breast carcinoma in the left breast. (B) Shows metastasis from right cervical lymph node. Scale bar represents 100 μm.

In November 2015, the patient was admitted to the hospital mainly due to finding a mass in her right neck, dysuria, and pain in her lower back. A cervical lymph node biopsy showed metastatic nodules (Fig. 1B). Immunohistochemistry: estrogen receptor (+ /-), progesterone receptor (-),human epidermal growth factor receptor 2 (+), basal cytokeratin 56 (CK56) (-), Thyroid transcription factor-1 (-),basal cytokeratin 7 (+) (Table 1). A bone scan showed multiple metastases of the sternum, multiple thoracic vertebrae of the spine, lumbar vertebra, multiple bilateral ribs, right scapula, pelvis, and right femur. In the L1 level the spinal canal narrowed down, the spinal cord cone was compressed and edema was present. After the fourth progress, lumbosacral local radiotherapy (30Gy/3Gy/10f) was given, and the endocrine drug Anastrozole was administered. After positron emission tomography - computed tomography on December 22, 2015, disease progression was found. Docetaxel + Capecitabine were used for 6 cycles of treatment. Later, multiple tumor markers showed a declining trend after reexamination (Table 3). Following that, SD was evaluated and the single drug Capecitabine was maintained.

**Table 3:**
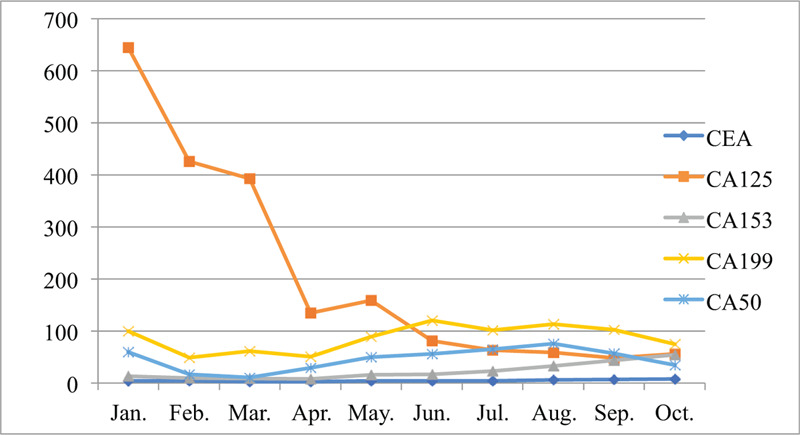
Changes of tumor markers in 2015. This change occurred 6 cycles after endocrine therapy was changed to chemotherapy.

CA = carbohydrate antigen, CEA = carcinoembryonic antigen.

In October 2016, the patient was admitted to the hospital due to an aggravated migraine lasting 1 week. The response evaluation criteria in solid tumors was SD. In February 2017, she was admitted to the hospital mainly due to night sweats and weakness of the lower right limb, accompanied by lumbar discomfort. The positron emission tomography - computed tomography showed a large amount of pleural effusion on the right side, and that the vertebral body was involved in a larger range than before, involving T12-L1. Fulvestrant was administered.

**Figure 2:**
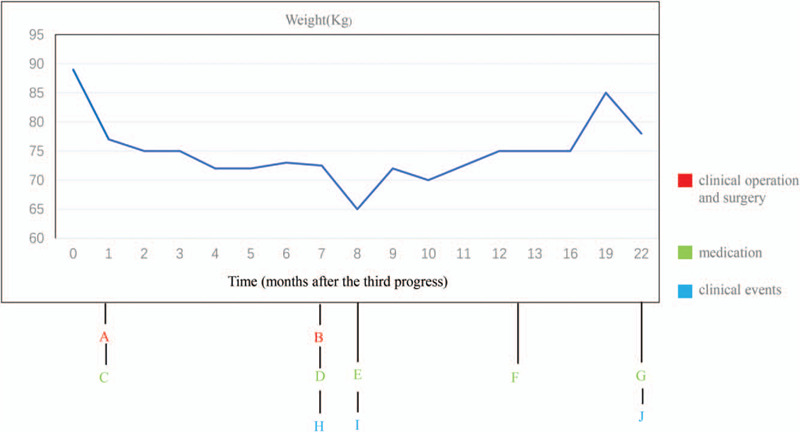
Weight versus time (months after the third progress) and surgical, pharmacological, and clinical events. Initial weight loss corresponds to clinical operation and treatment; subsequently, there was a sharp drop in weight at about 7 months, which was associated with the timing of several interventions and clinical events. The letters below the axis correspond to events, and the first line is clinical operation and surgery: (A) thoracic puncture and drainage, (B) cervical lymph node biopsy. The second line is medication: (C) stop letrozole and taxus chinensis, start Exemestane+ Goserelin, (D) lumbosacral local radiotherapy (30Gy/3Gy/10f), (E) start docetaxel + capecitabine, (F) start single drug capecitabine, and (G) start Fulvestrant, combined with radiofrequency deep hyperthermia of the tumor. The third line is clinical events: (H) in the L1 level spinal canal narrowed down, with spinal cord cone compressed and edema, (I) increased bone metastases, (J) the larger range of vertebral involvement than before.

Her course of illness was notable by significant weight loss (Fig. 2), a major indicator of the severity of disease. Over time, her weight loss could be affected by clinical events, medical interventions, or surgical interventions. In March 2017, she underwent thoracic laminectomy, tumor resection and pedicle screw fixation due to pain and numbness in her left lower limb. She weighed only 50 kg when she was admitted to the hospital on July 23, 2018.

Metabolic dysfunction leads to clinical deterioration in patients with advanced tumors. Weight loss, skeletal muscle wasting, and atrophy of the adipose tissue are common features. This systemic syndrome, termed cancer-associated cachexia (CAC), is a main cause of morbidity and mortality.^[6]^ Fortunately, we found a potential association between the growth of our patient's metastatic tumor burden and her cachexia. We followed in the footsteps of Consul et al, and quantified changes over time in the cross-sectional area of the following 5 tissue types at the L2 axial level: TU at the vertebral spine, subcutaneous fat (SF), visceral fat (VF), abdominal muscle (AM), and paraspinal muscle (PM).^[7]^

## 3 Methods

### 3.1 Histology

Biopsy tissues were fixed in 10% buffered formalin, embedded in paraffin, and cut into 5 μm sections on a Leica microtome. Histological analyses of all biopsy samples were performed by a pathologist (Table 1).

### 3.2 Radiological quantification

Cross-section at L2 vertebra. TU (Fig. 3 A), AM (Fig. 3 B), and PM (Fig. 3 C) cross-sectional areas were quantified within axial images of serial computational tomography abdomen/pelvis scans at a superior and an inferior L2 vertebral level. All cross-sectional areas were measured by a computational tomography radiologist using professional software.

**Figure 3:**
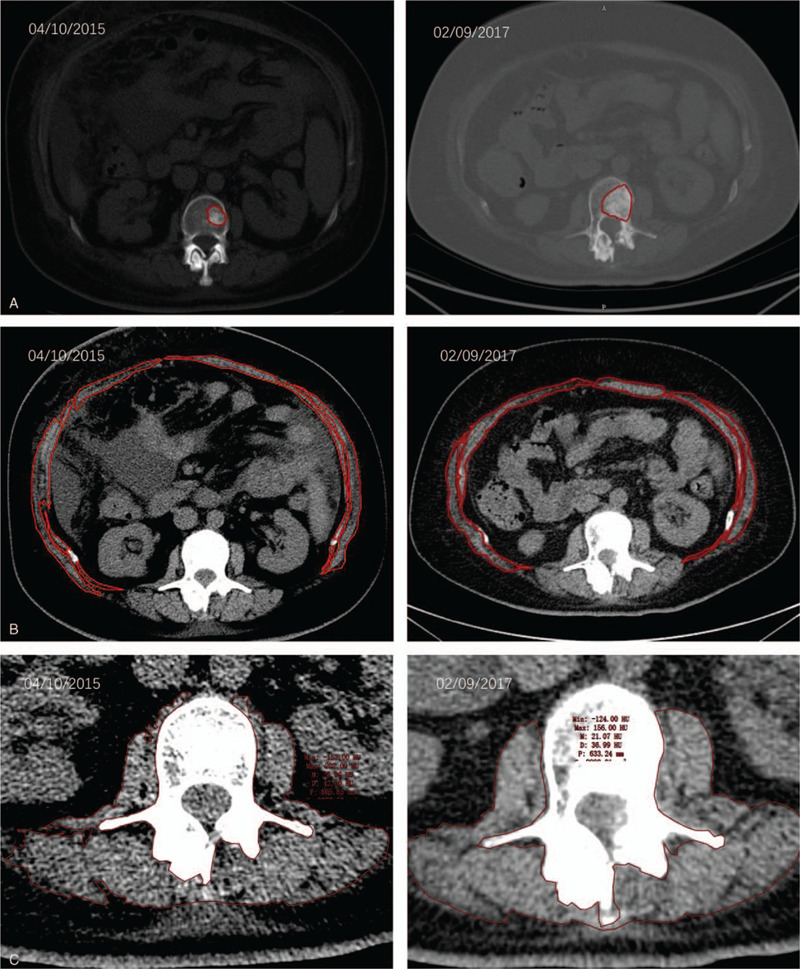
(A) TU segmentation. CT abdomen scan image at the L2 vertebral level. (B) AM segmentation. CT abdomen scan image at the L2 vertebral level. (C) PM segmentation. CT abdomen scan image at the L2 vertebral level. (D) Fat segmentation with top row of figures showing segmentation contours, and bottom row showing coloration of VF within the inside red contour and SF between the red contours. CT abdomen scan image at the L2 vertebral level.

**Figure 3 (Continued):**
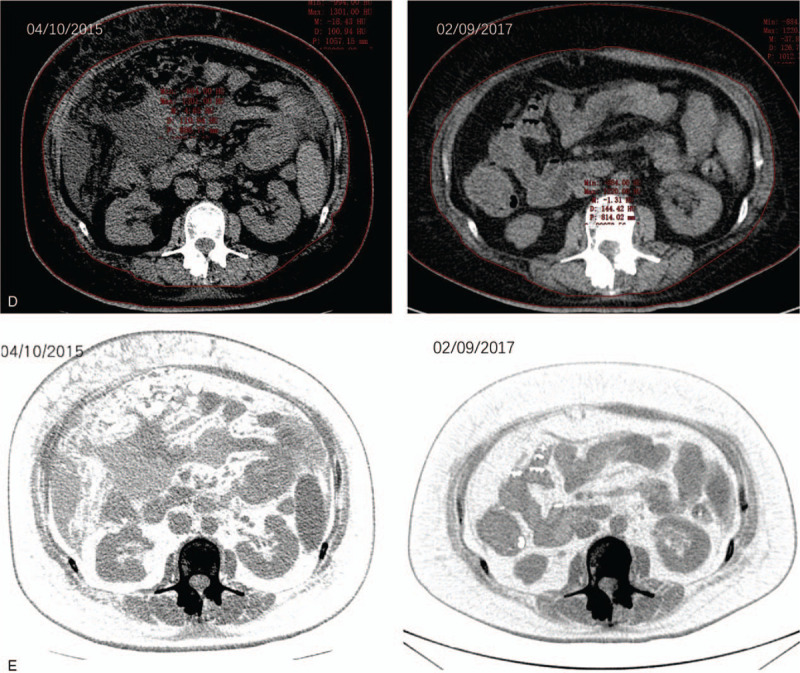
(A) TU segmentation. CT abdomen scan image at the L2 vertebral level. (B) AM segmentation. CT abdomen scan image at the L2 vertebral level. (C) PM segmentation. CT abdomen scan image at the L2 vertebral level. (D) Fat segmentation with top row of figures showing segmentation contours, and bottom row showing coloration of VF within the inside red contour and SF between the red contours. CT abdomen scan image at the L2 vertebral level.

**Figure 4:**
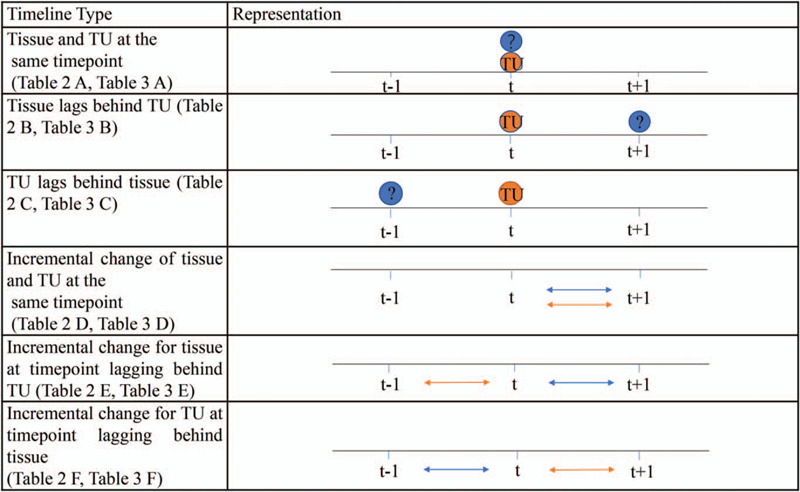
Visual representation of various timelines across which correlations were made. A description of the timepoint at which the tissue type (SF, VF, AM, PM) was used in a correlation with TU at the timepoint t is provided in the first column, with the corresponding correlation matrices listed in parentheses. A visual representation of the timeline shows when a tissue type (in blue) was measured and when TU (in red) was measured, before they were correlated to generate the correlation matrices in Tables 4 and 5.

SPSS 20.0 was used to calculate the Pearson (*r*) and Spearman (*ρ*) coefficients, and the correlation analysis was conducted on the original cross-sectional area data of each tissue type. These coefficients were used to generate correlation matrices (Tables 4 and 5). Compared with tumor burden, different time scales were used for different tissue types to assess the time delay that changes in one tissue type might present relative to another tissue type (Fig. 4). Microsoft Word was used to conditionally format the highest (pale yellow) and the lowest (bright green) values to distinguish associations between results for different tissue types.

**Table 4:** Pearson correlations (r) among all tissue types: TU, SF, VF, AM, and PM.

A	TU	SF	VF	AM	PM
TU	1.00	–0.60	–0.21	–0.66	–0.38
SF	–0.60	1.00	–0.10	–0.63	–0.46
VF	–0.21	–0.10	1.00	0.57	0.64
AM	–0.66	–0.63	0.57	1.00	0.25
PM	–0.38	–0.46	0.64	0.25	1.00

B	TU	SF	VF	AM	PM
TU	1.00	0.72	0.88	0.048	–0.73
SF	0.72	1.00	0.58	–0.54	–0.27
VF	0.88	0.58	1.00	0.114	–0.50
AM	0.048	–0.54	0.114	1.00	–0.58
PM	–0.73	–0.27	–0.50	–0.58	1.00

C	TU	SF	VF	AM	PM
TU	1.00	0.656	0.737	–0.948	–0.484
SF	0.656	1.00	–0.142	–0.86	–0.10
VF	0.737	–0.142	1.00	0.566	0.835
AM	–0.948	–0.86	0.566	1.00	0.36
PM	–0.484	–0.10	0.835	0.36	1.00

D	TU	SF	VF	AM	PM
TU	1.00	–0.147	0.255	–0.273	0.46
SF	–0.147	1.00	0.512	–0.597	0.755
VF	0.255	0.512	1.00	0.115	0.900
AM	–0.273	–0.597	0.115	1.00	–0.494
PM	0.46	0.755	0.900	–0.494	1.00

E	TU	SF	VF	AM	PM
TU	1.00	–0.784	0.592	0.968	–0.638
SF	–0.784	1.00	–0.22	–0.726	0.544
VF	0.592	–0.22	1.00	0.421	0.190
AM	0.968	–0.726	0.421	1.00	–0.802
PM	–0.638	0.544	0.190	–0.802	1.00

F	TU	SF	VF	AM	PM
TU	1.00	0.239	–0.518	–0.842	0.028
SF	0.239	1.00	0.522	–0.723	0.857
VF	–0.518	0.522	1.00	0.109	0.827
AM	–0.842	–0.723	0.109	1.00	–0.467
PM	0.028	0.857	0.827	–0.467	1.00

A box in the table holds the value of *r* that corresponds to the data for the 2 tissue types corresponding with the row and column assigned to that box. The strongest hue of green indicates a more negative correlation, while the weakest hue (pale yellow) indicates a more positive correlation. Looking at green colorations, the strongest negative correlations seem to exist between TU and another tissue type, along the first row or the first column. (A) Represents correlations for the cross-sectional raw areas as they were quantified and recorded. AM and TU are the most negatively correlated, with moderate negative correlations of SF against TU. (B) Represents correlations for areas of TU at the timepoint t against the areas of the other 4 tissue types at the timepoint t + 1. This shows that PM against TU are most negatively correlated, with moderate positive correlations of SF against VF. (C) Represents correlations for areas of TU at the timepoint t against the areas of the other four tissue types at the timepoint t − 1. AM against TU are negatively correlated. (D) Represents correlations among the incremental gains in the area for each tissue type during an interval between the same timepoints. Here, there is a strong positive correlation of PM against VF. (E) Represents the correlation between the incremental gain in the area for TU at the timepoint t against the incremental gain in areas for the other four tissue types at the timepoint t + 1. This shows a moderate negative correlation of PM against TU and SF against TU. (F) Represents the correlation between the incremental gain in the area for TU at the timepoint t against the incremental gain in areas for the other 4 tissue types at the timepoint t − 1. There is a strong negative correlation of AM against TU, with a moderate positive correlation of SF against VF. AM = abdominal muscle, PM = paraspinal muscle, SF = subcutaneous fat, TU = tumor burden at vertebral spine, VF = visceral fat.

**Table 5:** Spearman correlations (ρ) among all tissue types: tumor, SF, VF, AM, and PM.

A	TU	SF	VF	AM	PM
TU	1.00	0.486	–0.03	–0.71	–0.37
SF	0.486	1.00	0.26	–0.43	–0.26
VF	–0.03	0.26	1.00	0.37	0.37
AM	–0.71	–0.43	0.37	1.00	–0.03
PM	–0.37	–0.26	0.37	–0.03	1.00

B	TU	SF	VF	AM	PM
TU	1.00	0.70	0.80	–0.10	–0.40
SF	0.70	1.00	0.70	–0.10	–0.10
VF	0.80	0.70	1.00	–0.10	–0.10
AM	–0.10	–0.10	–0.10	1.00	–0.80
PM	–0.40	–0.10	–0.10	–0.80	1.00

C	TU	SF	VF	AM	PM
TU	1.00	0.60	0.40	–1	–0.10
SF	0.60	1.00	0.30	–0.60	0.30
VF	0.40	0.30	1.00	0.40	0.70
AM	–1	–0.60	0.40	1.00	0.10
PM	–0.10	0.30	0.70	0.10	1.00

D	TU	SF	VF	AM	PM
TU	1.00	0	0.50	–0.60	0.60
SF	0	1.00	0.50	–0.20	0.70
VF	0.50	0.50	1.00	0.10	0.803
AM	–0.60	–0.20	0.10	1.00	–0.30
PM	0.60	0.70	0.803	–0.30	1.00

E	TU	SF	VF	AM	PM
TU	1.00	–0.80	0.40	0.80	–0.20
SF	–0.80	1.00	0	–0.40	0.40
VF	0.40	0	1.00	0.20	0.80
AM	0.80	–0.40	0.20	1.00	–0.40
PM	–0.2	0.40	0.80	–0.40	1.00

F	TU	SF	VF	AM	PM
TU	1.00	0.20	–0.40	–0.80	0.20
SF	0.20	1.00	0.80	–0.40	1
VF	–0.40	0.80	1.00	0.20	0.80
AM	–0.80	–0.40	0.20	1.00	–0.40
PM	0.20	1	0.80	–0.40	1.00

Abox in the table holds the value of the Spearman correlation coefficient that corresponds to the data for the 2 tissue types corresponding with the row and column assigned to that box. The strongest hue of green indicates a more negative correlation, while the weakest hue (pale yellow) indicates a more positive correlation. Looking at green colorations, the strongest negative correlations seem to exist between TU and another tissue type, along the first row or the first column. (A) Represents correlations for the cross-sectional raw areas as they were quantified and recorded. TU and AM are the most negatively correlated, with little correlations of the other tissues against TU. (B) Represents correlations for areas of TU at the timepoint *t* against the areas of the other 4 tissue types at the timepoint *t* + 1. This shows VF against TU is the most strongly positively correlated. (C) Represents correlations for areas of TU at the timepoint *t* against the areas of the other 4 tissue types at the timepoint *t* − 1. VF against TU and AM against TU are completely negative correlated. (D) Represents correlations among the incremental gains in the area for each tissue type during an interval between the same timepoints. Here, there is a moderate negative correlation of AM against TU, with a moderately/strongly positive correlation of VF against SF and VF against PM. (E) Represents the correlation between the incremental gain in the area for TU at the timepoint *t* against the incremental gain in areas for the other 4 tissue types at the timepoint *t* + 1. This shows a strong negative correlation of SF against TU and a strong negative correlation of VF against PM. (F) Represents the correlation between the incremental gain in the area for TU at the timepoint *t* against the incremental gain in areas for the other 4 tissue types at the timepoint *t* − 1. There is a strong negative correlation of AM against TU, with a strong positive correlation of PM against VF. AM = abdominal muscle, PM = paraspinal muscle, SF = subcutaneous fat, TU = tumor burden at vertebral spine, VF = visceral fat.

## 4 Results

### 4.1 Pathology

A biopsy of breast tissue from the left breast revealed invasive ductal carcinoma consistent with the previously mentioned clinical diagnosis (Fig. 1A). Tissue samples from the right cervical lymph node (Fig. 1B) revealed metastases of the breast carcinoma in the other site. A radionuclide bone scan revealed multiple systemic bone metastases, including sternum, multiple thoracic vertebrae of the spine, lumbar vertebra, and multiple bilateral ribs.

### 4.2 Radiological quantification: cross-section at L2 vertebra

Tumor tissue segmentation revealed an overall increase in TU from April 10, 2015, to February 09, 2017 (Fig. 3 A), while AM and PM segmentations revealed an overall decrease from April 10, 2015, to February 09, 2017 (Fig. 3 B and C, respectively). Fat segmentation and segregation into VF and SF also revealed an overall decrease in both SF and VF from April 10, 2015, to February 09, 2017 (Fig. 3 D). Measurements of cross-sectional tissue areas plotted over time revealed opposing trends in the areas of PM, AM, VF, and SF versus the area of TU (Tables 6 and 7), further investigated with correlation analysis. Correlation matrices of *r* are shown in Table 4A–F, and correlation matrices of ρ are shown in Table 5A–F. The matrices were built to show correlation coefficients of 1.00 when a tissue type is correlated with itself. Correlations among the raw cross-sectional areas of the 5 tissue types generally showed weak positive correlations among SF, VF, AM, and PM, but showed weak-to-strong negative correlations when any of SF, VF, AM, or PM was correlated with TU.

**Table 6:**
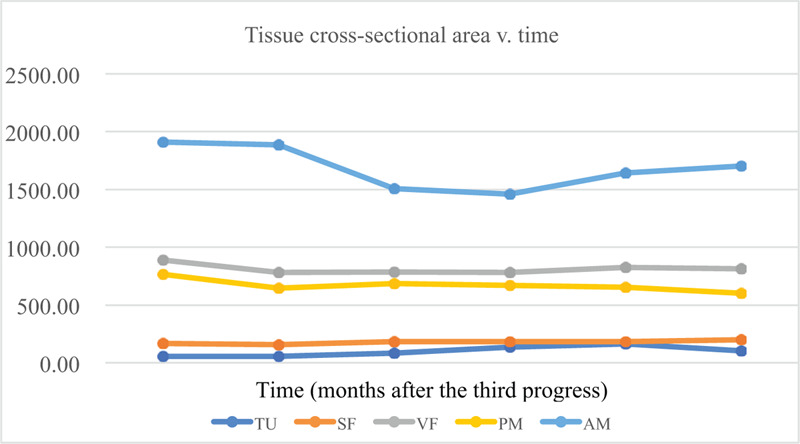
Tissue cross-sectional area versus time.

**Table 7:**
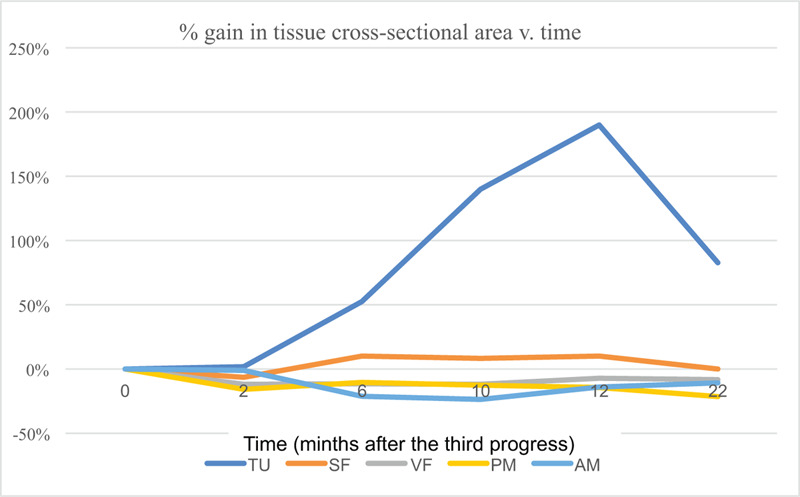
Percentage gain in tissue cross-sectional area versus time.

Comparing multiple different types of tissue growth (TU, SF, VF, AM, and PM) over time reveals potential trends when normalized to percent gain; while when utilizing raw data, trends are more difficult to see as each tissue cross-sectional area is on a different order of magnitude of scale. (B) Percentage gain in tissue cross-sectional area versus time. Comparing multiple different types of tissue growth (TU, SF, VF, AM, and PM) over time reveals potential trends when normalized to percent gain; while when utilizing raw data, trends are more difficult to see as each tissue cross-sectional area is on a different order of magnitude of scale. AM = abdominal muscle, PM = paraspinal muscle, SF = subcutaneous fat, TU = tumor burden at vertebral spine, VF = visceral fat

### 4.3 SF versus TU

SF had a strong negative correlation against TU with *r* = –0.60 (Table 6), *r* = –0.784 (Table 4E), ρ = –0.80 (Table 5E). Negative correlations for SF and TU, with SF at the lagging timepoint compared to TU, indicates that decreases in SF occurred after a time lag following increases in TU in our case study patient (Fig. 4). However, the negative correlation between SF and TU is generally not significant. (Table 7).

### 4.4 VF versus TU

VF correlated weakly negatively against TU with *r* = –0.518 (Table 4F). Negative correlations for VF and TU, with VF at the timepoint preceding TU, indicates that decreases in VF preceded increases in TU that occurred after a time lag, and vice versa (Fig. 4). This corresponds with valleys in the graph of VF over time at timepoints just prior to the peak in TU (Table 7).

### 4.5 AM versus TU

AM correlated strongly and negatively against TU with *r* = –0.66 (Table 6), *r* = –0.948 (Table 4C), *r* = –0.84 (Table 4F), ρ = –0.71 (Table 5A), ρ = –1 (Table 5C), ρ = –0.60 (Table 5D) and ρ = –0.80 (Table 5F). Correlations between AM and TU were moderately negative when the incremental change in AM was compared to the incremental change in TU between the same 2 timepoints. Negative correlations between AM and TU were strong when AM at the preceding timepoint was compared to TU at the following timepoint, indicating that changes in AM preceded the inverse change in TU that occurred after a time lag, and vice versa (Fig. 4). As in the line graph, AM tends to move in the opposite direction of TU, and the valley value of AM is exactly 1 point ahead of the peak of TU (Table 7).

### 4.6 PM versus TU

PM correlated moderately negative against TU with *r* = –0.73 (Table 7) and *r* = –0.638 (Table 4E). Negative correlations for PM and TU, with PM at the lagging timepoint compared to TU, indicates that decreases in PM occurred after a time lag following increases in TU in our case study patient (Fig. 4). Notably, AM, PM, and VF experienced few increases throughout its growth over the 2-year period assessed, while SF experienced remarkable increases and decreases over time (Table 7).

In short, VF and AM correlate negatively with TU when TU is measured at a lagging timepoint, and PM correlates negatively with TU when assessed at a lagging timepoint as compared to TU. In this case, the correlation between SF and TU is not prominent.

## 5 Discussion

Although cancer-related cachexia is often attributed to loss of appetite and the side effects of chemotherapy, it may be closely associated with the destruction of normal tissue by tumor secretory factors and clinical events or therapeutic interventions. Our patient with advanced breast cancer had severe cachexia. The mechanism by which cachexia promotes tumor invasion and metastasis is not yet clear, but it may be related to the inflammatory response, hypoxic state, decreased leptin levels and release of pro-angiogenic factors in cachexia patients.^[8]^ Consequently, a vicious cycle occurs, with malnutrition from insufficient dietary intake resulting in increasing therapy toxicity and the unmanaged tumor causing even more severe malnutrition.

As we focus on weight loss, we attempt to define cachexia more comprehensively through body composition, to be incorporated into routine clinical practice.^[9]^ According to a study conducted by Shen et al, the area of muscle/fat in a cross section of the abdomen best represents the total volume of muscle/fat in the entire body.^[10]^

In our case study, the overall weight of our patient remained stable at times during the 22 months of the study. It would sometimes drop or rise rapidly, but the metastasis continued to progress (Fig. 2), and AM loss continued (Fig. 3 ). The total area of SF, VF, AM, and PM decreased, resulting in a poor prognosis. Finally, the increased tumor burden and the decreased in the total area of SF, VF, AM, and PM were taken as the model (Fig. 3 ).^[11]^

In our case study, we chose the level of the second lumbar vertebra, because the metastases here were ongoing and clear from beginning to end. The increasing tumor burden at L2, associated with advanced-stage cancer, correlated with the area of other tissues at L2. The 5 tissues were statistically correlated to explore which tissue (muscle or fat) best predicted tumor progression. Surprisingly, the correlation between AM and TU at L2 is strongly negative when the raw area or incremental gains in AM at a specific timepoint is compared to TU at a latter timepoint. This mean that any change in TU tended to correlate strongly with the change in AM that occurred at the previous timepoint (Tables 4C and F and 5C and F). Although SF, VF, and PM also correlated negatively with TU across different timelines, VF did not decrease significantly during the entire observation process and was often higher than the initial value. AM, PM, and SF continuously decreased over time, but the patterns of change were different. Polyline PM continued to decrease regardless of the increase or decrease of TU, but polyline AM and SF decreased when TU continued to increase, while the decrease rate declined before TU decreased. Since these hypotheses were extracted from data on a single patient, no causal relationship can be drawn and extrapolations for all breast cancer patients are unfounded. In addition, we cannot exclude other factors that may lead to comorbidities, such as side effects of chemotherapy manifested as anorexia and inadequate intake of protein and fat, resulting in weight loss, and AM and PM reduction. However, in our patient and the patient of Consul et al,^[7]^ the 5 tissue areas on the L2 cross-section illustrate the relationship. This can be studied in future multi-patient- controlled studies.

Imaging quantifications suggest that the strongest negative Pearson correlation among our patient's 5 tissue types was between the area of AM at a preceding timepoint and the area of TU, correlated with *r* = –0.948 (Table 4C). The strongest negative Spearman correlation was between the area of AM at a preceding timepoint and the area of TU, correlated with *ρ* = –1 (Table 5C). SF correlated negatively or positively with TU depending on how they were compared across time. VF and TU were weakly negatively correlated with VF at a preceding timepoint, but PM and TU were moderately negatively correlated with PM at a lagging timepoint These findings indicate that the mechanisms behind VF and AM loss may proceed more rapidly than those that govern the increase in TU. PM loss may proceed slower than those that govern the increase in TU. Finally, the SF loss had little to do with the increasing TU. We considered whether AM loss may be correlated with progressive bone metastasis, and found that the change curve of AM is the most consistent with the fluctuation of TU.

Recent evidence suggests that factors released by bone and muscle interact. The example discussed here, bone metastases, represents a severe disruption of normal bone remodeling. Bone is a rich storehouse of growth factors that have activity in the bone (as a part of normal remodeling) and in other organs, including muscle. It is therefore possible that during accelerated bone resorption, such as that which occurs in bone metastases, bone might have a predominant role in altering muscle function and becoming a source of “osteokines” that affect muscle function. Likewise, factors released from muscle may have an important role in bone metabolism that could further exacerbate the role of bone as a driver of muscle dysfunction. Whatever factors are identified that transmit signals between bone and muscle, it is clear that bone-derived factors are capable of impacting muscle and that the effects can manifest as a reduction in muscle mass (quantity), myocyte function (quality) or both, as is likely in advanced disease.^[12]^ Although cancer cachexia is a wasting syndrome characterized by muscular atrophy with systemic inflammation, when considering the relationship between weight loss and cachexia, fat loss that occurs before muscle wasting must be considered. Sarcopenia was radiologically evident in the AM; therefore, an increase in weight could be due to an increase in fat content. The continuous decline in PM could describe an underlying relationship between muscle mass and the adjacent site of bony metastatic bone disease. We would like to explore the relationship between bone metastasis and AM reduction further.

Given the influential role of cachexia in the clinical presentation of breast cancer patients, yet low documentation thereof, it is crucial to overcome the problem of its underdiagnosis in clinical practice. Early diagnosis of cachexia via careful monitoring of muscle wasting, perhaps through routine radiological studies, could allow for earlier intervention and therefore prevention of energy deprivation. Furthermore, recognizing the interrelationship between muscle wasting and tumor metastasis to the skeletal system as an important mechanism behind the rise of cachexia in breast cancer is a step toward building new methods for early diagnosis of metastasis. We believe early nutritional support may reduce muscle and fat loss, improve cachexia, and delay the progression of bone metastasis. Meanwhile, several studies have identified an association between the pre-cachectic status and better treatment response.

This is a single case study. More research is needed to further reveal whether the correlation between AM and TU described in this case will exist in most advanced breast cancer patients. We should also analyze multiple similar cases to explore which area of muscle is most suited as a prognostic factor of cachexia and tumor metastasis. And we can rule out other factors that can cause comorbidity.

## 6 Conclusion

Although there are few cases of cachexia in breast cancer, our patients had muscle loss through bone metastasis and the interaction between muscles, meeting the criteria of cachexia. According to our data and references, the overall trend of AM was consistent with that of TU at the level of L2, and the change of AM occurred earlier than that of TU. Therefore, the longitudinal monitoring of cachexia can select an abdominal muscle group to provide a method for clinicians to judge the degree and prognosis of breast cancer cachexia in the early stage.

## Acknowledgments

We thank the contributions of the imaging department and nuclear medicine department to this study, especially Xiaojuan Tian. We thank Dr. Yuanfei Li for advice and support.

## Author contributions

**Conceptualization:** Yuanfei Li.

**Data curation:** Nan Liu, Ying Qiao.

**Formal analysis:** Nan Liu, Shuting Li.

**Investigation:** Nan Liu, Shuting Li, Yuanfei Li.

**Methodology:** Nan Liu, Shuting Li, Ying Qiao.

**Project administration:** Yuanfei Li.

**Resources:** Junmei Jia, Ying Qiao, Yuanfei Li.

**Software:** Nan Liu, Shuting Li.

**Supervision:** Junmei Jia, Ying Qiao, Yuanfei Li.

**Visualization:** Nan Liu.

**Writing – original draft:** Nan Liu.

**Writing – review & editing:** Nan Liu, Shuting Li, Junmei Jia, Ying Qiao, Yuanfei Li.

## Abbreviations

Abbreviations: *ρ* = Spearman correlation coefficient, AM = abdominal muscle, PM = paraspinal muscle, *r* = Pearson correlation coefficient, SD = stable disease, SF = subcutaneous fat, TU = tumor burden at vertebral spine, VF = visceral fat.

## References

[1] Spano D Heck C De Antonellis P. Molecular networks that regulate cancer metastasis. Semin Cancer Biol 2012;22:234–49. 22484561 10.1016/j.semcancer.2012.03.006

[2] Tisdale MJ. Cancer cachexia. Curr Opin Gastroenterol 2010;26:146–51. 19918173 10.1097/MOG.0b013e3283347e77

[3] Fearon KC. Cancer cachexia: developing multimodal therapy for a multidimensional problem. Eur J Cancer 2008;44:1124–32. 18375115 10.1016/j.ejca.2008.02.033

[4] Blauwhoff-Buskermolen S Langius JAE Becker A. The influence of different muscle mass measurements on the diagnosis of cancer cachexia. J Cachexia Sarcopenia Muscle 2017;8:615–22. 28447434 10.1002/jcsm.12200 PMC5566652

[5] Xu WW Wang SA Zou ZY. The occurrence and development of cachexia and muscle microenvironment. Electron J Metab Nutr Cancer 2017;4:247–52.

[6] Fielding RA Vellas B Evans WJ. Sarcopenia: an undiagnosed condition in older adults. Current consensus definition: prevalence, etiology, and consequences. International working group on sarcopenia. J Am Med Dir Assoc 2011;12:249–56. 21527165 10.1016/j.jamda.2011.01.003 PMC3377163

[7] Consul N Guo X Coker C. Monitoring metastasis and cachexia in a patient with breast cancer: a case study. Clin Med Insights Oncol 2016;10:83–94. 27660506 10.4137/CMO.S40479 PMC5019129

[8] Shen ST Zheng ZW Liao ZK. Molecular mechanisms of cachexia and tumor metastasis. Electron J Metab Nutr Cancer 2018;5:134–8.

[9] Bruggeman AR Kamal AH LeBlanc TW. Cancer cachexia: beyond weight loss. J Oncol Pract 2016;12:1163–71. 27858548 10.1200/JOP.2016.016832

[10] Shen W Punyanitya M Wang Z. Total body skeletal muscle and adipose tissue volumes: estimation from a single abdominal cross-sectional image. J Appl Physiol (1985) 2004;97:2333–8. 15310748 10.1152/japplphysiol.00744.2004

[11] Baracos VE Reiman T Mourtzakis M. Body composition in patients with non-small cell lung cancer: a contemporary view of cancer cachexia with the use of computed tomography image analysis. Am J Clin Nutr 2010;91:1133S–7S. 20164322 10.3945/ajcn.2010.28608C

[12] Waning DL Guise TA. Cancer-associated muscle weakness: What's bone got to do with it? Bonekey Rep 2015;4:1–6. 10.1038/bonekey.2015.59 PMC4432780 25992285

